# Effect on the Aroma Profile of *Graciano* and *Tempranillo* Red Wines of the Application of Two Antifungal Treatments onto Vines

**DOI:** 10.3390/molecules190812173

**Published:** 2014-08-13

**Authors:** Raquel Noguerol-Pato, Thais Sieiro-Sampredro, Carmen González-Barreiro, Beatriz Cancho-Grande, Jesús Simal-Gándara

**Affiliations:** Group of Nutrition and Bromatology, Department of Analytical and Food Chemistry, Faculty of Food Science and Technology, University of Vigo, Ourense E-32004, Spain

**Keywords:** boscalid, kresoxim-methyl, metrafenone, odour activity value (OAV), odorant series, Graciano and Tempranillo red wines

## Abstract

The effect of two antifungals (boscalid + kresoxim-methyl and metrafenone) applied onto vines under Good Agricultural Practices (GAPs) on the volatile composition of Tempranillo and Graciano red wines was studied. Changes in aroma profile in the wines were assessed from the combined odour activity values (OAVs) for the volatile compounds in each of seven different odorant series (viz., ripe fruits, fresh fruits, lactic, floral, vinous, spicy and herbaceous). Graciano wines obtained from grapes treated with the antifungals exhibited markedly increased concentrations of varietal volatile compounds (monoterpenes and C_13_-norisoprenoids) and aldehydes, and decreased concentrations of acetates and aromatic alcohols. By contrast, the concentrations of volatile compounds in Tempranillo wines showed different changes depending on the fungicide applied. Also, the aroma profiles of wines obtained from treated grapes were modified, particularly the ripe fruit nuances in Graciano wines. The OAV of this odorant series underwent an increase by more than 60% with respect to the control wine as a result of the increase of β-damascenone concentration (which imparts wine a dry plum note). The aroma profile of Tempranillo red wines containing metrafenone residues exhibited marked changes relative to those from untreated grapes.

## 1. Introduction

Wine grapes are susceptible to a large number of stresses in most production areas. One of the most significant, in terms of economic losses, are fungal diseases (especially, grey mould, powdery mildew and downy mildew) which are mainly combated with fungicide application. Plants have the ability to respond to fungicide treatments in many ways that include changes in photosynthetic rates [1], production of phytochemicals [2], pigment concentrations [3], and many other aspects of plant growth and regulation [4,5]. Such changes may have an effect on the sensory quality of wines produced from treated grapes, especially on the aroma profile which is particularly relevant as is one of the most appreciated characteristics in wines.

In this sense, flusilazole treatments seemed to influence the biosynthesis of some free and glycosidic volatile compounds in Muscat of Alexandria grapes [6] and, as a result, their levels in the obtained wines [7]. However, most variations in the aroma profile of wines related to fungicide applications are attributed to alterations in the growth and metabolism of yeasts during alcoholic and malolactic fermentation. After fungicide treatments, even when GAPs are followed, fungicide residues may remain on grapes and be transferred to the must and wine during winemaking [8,9,10,11,12,13] where they may alter yeast growth and metabolism [12,14,15,16,17,18] and, hence, the biosynthesis of volatile compounds and their concentrations in the final wine. In this context, Oliva *et al.* [19] evaluated the influence of pesticide residues on the aromatic composition of red wines made from *Vitis vinifera* var. Monastrell grapes and found that significant differences with respect to control wines were detected in the concentration of several fermentative volatile compounds such as isopentyl acetate, ethyl acetate and 2-methyl-1-propanol. Also, it was observed by Oliva *et al.* [20] that the application of famoxadone, fenhexamid, fluquinconazole, kresoxim-methyl and trifloxystrobin, under GAPs, and quinoxyfen, kresoxim-methyl, fluquinconazole and trifloxystrobin, under Critical Agricultural Practices (CAPs) significantly affected the aroma composition of Monastrell red wines. González-Rodríguez *et al.* [21] observed that the application of several fungicides, such as benalaxil, iprovalicarb and pyraclostrobin, under GAPs, seemed to cause an increase in the levels of several ethyl esters and acetates of Godello white wines. After that, González-Álvarez *et al.* [22] continued with the study of the effect of other fungicides (cyazofamid, famoxadone, mandipropamid and valifenalate, applied under CAPs) on the aromatic content of Godello white wines. In this case, mainly fermentative volatile compounds (esters and acids) were affected by the presence of fungicide residues. Noguerol-Pato *et al.* [23] examined the effect of tebuconazole residues on the aromatic composition of Mencía red wines obtained by using endogenous and commercial yeasts to carry out the alcoholic fermentation. Recently, Noguerol-Pato *et al.* [17] observed that the synthesis of ethyl esters and acetates by *Saccharomyces cerevisiae* during the alcoholic fermentation was radically reduced in Tempranillo red wines in presence of ametoctradin, dimethomorph and mepanipyrim residues. Also, the addition of mepanipyrim and fenhexamid to Tempranillo and Graciano grape musts modified the aromatic profile of the obtained wines [24].

The main aim of this work was to study the effect of fungicides used against powdery mildew (*Erisiphe necator*, formerly *Uncinula necator*) and grey mould (*Botrytis cinerea*) on the volatile composition and aroma profile of Graciano and Tempranillo red wines elaborated from grapes treated under GAPs with boscalid+kresoxim-methyl and metrafenone, separately.

## 2. Results and Discussion

### 2.1. Effect of Application under GAPS of Boscalid + Kresoxim-Methyl and Metrafenone on the Volatile Composition of Graciano and Tempranillo Red Wines

A total of 42 and 43 volatile compounds were determined and quantified in Tempranillo and Graciano wines, respectively, including four monoterpenes, two C_13_-norisoprenoids, nine alcohols, 11 esters, seven fatty acids, seven volatile phenols, two lactones and one aldehyde. Table 1 lists the concentrations and standard deviations of the volatile compounds grouped according to their chemical structure and origin in wine.

In general terms, aliphatic and aromatic alcohols (alcohols from the fermentative stage) constituted the largest fraction of volatile compounds in the wines, although to be more precise, only two volatile compounds (isoamyl alcohols and 2-phenylethanol) accounted for 90% of the total. The other chemical families of volatile compounds present in the wine, in decreasing order of abundance, were fatty acids, ethyl esters, C_6_ alcohols, acetates, volatile phenols, lactones, aldehydes, monoterpenes and C_13_-noriosoprenoids.

**Figure 1:**
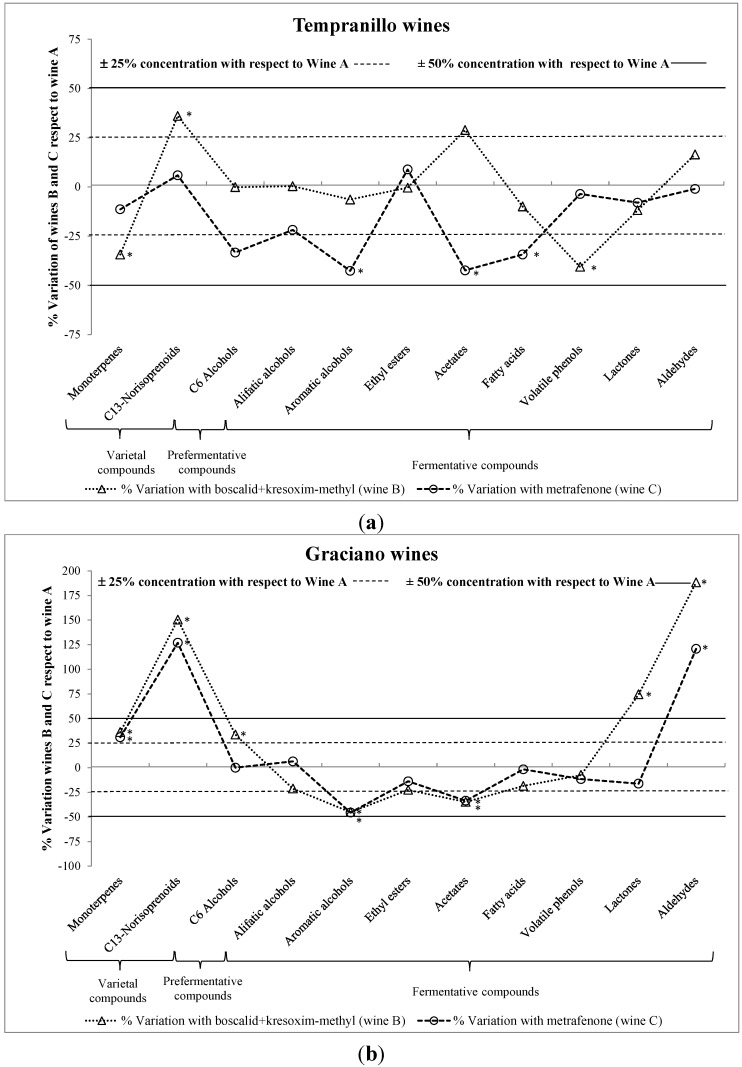
Percentages of variation with respect to control wine (A) on the total concentrations of volatile compounds: Tempranillo-based wines (**a**) and Graciano-based wines (**b**). Significant differences at *p* < 0.01 (Fisher’s least significant difference test) with respect to control wines were pointed out with an asterisk (*).

Figure 1a,b show the percentages of variation in overall concentration of each chemical family in wines B and C with respect to the control (wine A). Concentration of varietal compounds in wines (monoterpenes and C_13_-norisoprenoids) showed different behaviors depending on the grape variety and fungal treatment. In Tempranillo-based wines, the concentrations of monoterpenes and C_13_-norisoprenoids barely changed in wine C (metrafenone), however boscalid+kresoxim-methyl treatment (wine B) appeared to cause variations of up to 25% in the concentration of both groups with respect to control (wine A). Meanwhile, the application of boscalid+kresoxim-methyl and metrafenone to Graciano grapes seemed to lead to similar modifications in the concentrations of monoterpenes (about 30% higher) and C_13_-norisoprenoids (about 120% higher) in the obtained wines. Geraniol and β-citronellol exhibited the highest concentrations among monoterpenes, and β-damascenone among C_13_-norisoprenoids; therefore, these compounds were directly responsible for the differences between wines B–C and wine A. The general increase in the concentrations of varietal compounds can be explained by the stimulation of yeast glycosidase activity during alcohol fermentation which leads to a stronger release of these volatile compounds from their respective non-volatile structures [25]. However, since fungicides were applied during the vine growth, the fungal substances may have affected the monoterpene biosynthesis and the glycosylation pathways in the plant as Aubert *et al.* [6] suggested. In this respect, similar results were previously found by Noguerol-Pato *et al.* [24] in Tempranillo and Graciano wines when their musts were supplied with mepanipyrim and fenhexamid. Oliva *et al.* [20] also found increased concentrations of terpenoids (nerolidol and damascenone) in Monastrell red wines from grapes treated with kresoxim-methyl, famoxadone, fluquinconazole or fenhexamid. 

**Table 1:** Concentration of volatile compounds (μg/L) in Tempranillo and Graciano red wines after fungicide applications (mean values and SD, *n* = 3 replicates).

Volatile Compounds		Tempranillo Red Wines			Graciano Red Wines			Grape Variety × Fungicide Treatment
		Wine A ^a^	Wine B ^b^	Wine C ^c^	Wine A ^a^	Wine B ^b^	Wine C ^c^	*F*-values
**Varietal compounds**	**Monoterpenes**							
	(±)-Linalool	1.0 ± 0.061	1.4 ± 0.15	1.2 ± 0.14	2.5 ± 0.14	4.5 ± 0.68	4.2 ± 0.24	
	α-Terpineol	≤0.2	≤0.2	≤0.2	1.0 ± 0.061	1.2 ± 0.025	1.2 ± 0.088	
	(±)-β-Citronellol	11 ± 1.4	5.0 ± 0.66	11 ± 0.27	8.7 ± 0.37	12 ± 0.35	12 ± 0.43	
	Geraniol	8.8 ± 0.85	7.7 ± 0.70	6.6 ± 0.83	10 ± 0.92	13 ± 1.0	12 ± 0.78	
	***Sum of monoterpenes***	**21 ± 2.3 bd**	**14 ± 1.4 a**	**19 ± 1.1 ab**	**23 ± 1.2 a**	**31 ± 1.1 b**	**30 ± 1.3 b**	15.53 ^***^
	**C_13_-norisoprenoids**							
	β-Damascenone	1.4 ± 0.19	2.0 ± 0.19	1.5 ± 0.23	3.2 ± 0.045	8.4 ± 0.59	7.9 ± 0.28	
	β-Ionone	0.31 ± 0.025	0.35 ± 0.012	0.28 ± 0.0030	0.69 ± 0.033	1.4 ± 0.11	1.0 ± 0.00	
	***Sum of C_13_-norisoprenoids***	**1.7 ± 0.20 a**	**2.3 ± 0.18 b**	**1.8 ± 0.010 a**	**3.9 ± 0.061 a**	**9.8 ± 0.068 b**	**8.9 ± 0.28 b**	**1.24 ns**
**Pre-fermentative compounds**	**C_6_ alcohols**							
	1-Hexanol	2,534 ± 374	2,389 ± 98	1,583 ± 104	2,745 ± 189	3,611 ± 396	2,720 ± 133	
	*trans*-3-Hexen-1-ol	57 ± 4.3	62 ± 5.7	52 ± 6.5	58 ± 1.8	95 ± 8.4	76 ± 4.5	
	*cis*-3-Hexen-1-ol	225 ± 6.5	360 ± 16	241 ± 33	22 ± 1.3	66 ± 5.3	30 ± 1.78	
	***Sum of C_6_ alcohols***	**2,815 ± 387** **a**	**2,812 ± 119 a**	**1,888 ± 152 a**	**2,873 ± 197 a**	**3,773 ± 409 b**	**2,826 ± 128 a**	**0.00 ns**
**Fermentative compounds**	**Aliphatic alcohols**							
	2-Methyl-1-propanol	567 ± 58	516 ± 66	438 ± 40	545 ± 92	491 ± 41	642 ± 63	
	1-Butanol	44 ± 2.4	357 ± 18	414 ± 44	342 ± 67	389 ± 49	405 ± 6.2	
	Isoamyl alcohols	169,570 ± 12,312	169,948 ± 4,518	132,191 ± 16,528	133,225 ± 8,480	104,536 ±5,096	141,672 ± 24,804	
	1-Octanol	15 ± 1.6	15 ± 1.8	22 ± 0.36	4.2 ± 0.63	12 ± 0.10	9.1 ± 0.30	
	***Sum of aliphatic alcohols***	**170,196 ± 12,256 a**	**170,836 ± 4,469 a**	**133,226 ± 16,648 a**	**134,057 ± 8,631 a**	**105,431 ± 5,223 a**	**142,729 ± 24,754 a**	11.14 ^**^
	**Aromatic alcohols**							
	Benzyl alcohol	121 ± 11	82 ± 7.0	111 ± 15	816 ± 71	648 ± 3.9	983 ± 84	
	2-Phenylethanol	52,910 ± 7,178	49,544 ± 4,631	30,315 ± 2,328	53,576 ± 3,927	29,182 ± 4,317	28,644 ± 658	
	***Sum of aromatic alcohols***	**53,032 ± 7,169** **b**	**49,626 ± 4,636 ab**	**30,430 ± 2,347 a**	**54,393 ± 3,945 b**	**29,924 ± 4,481 a**	**29,642 ± 610 a**	19.81 ^***^
	**Ethyl esters**							
	Ethyl caproate	427 ± 65	526 ± 19	336 ± 48	287 ± 32	309 ± 12	331 ± 78	
	Ethyl lactate	2,800 ± 206	3,086 ± 167	2,799 ± 126	1,583 ± 227	1,246 ± 155	1,415 ± 52	
	Ethyl caprylate	495 ± 63	500 ± 41	330 ± 38	270 ± 22	238 ± 8.5	252 ± 5.9	
	Ethyl 3-hydroxybutyrate	905 ± 66	644 ± 61	1,139 ± 153	312 ± 13	290 ± 23	264 ± 12	
	Ethyl decanoate	44 ± 2.7	48 ± 3.6	50 ± 6.6	39 ± 0.71	43 ± 3.5	42 ± 2.5	
	Diethyl succinate	505 ± 42	340 ± 12	984 ± 59	651 ± 92	383 ± 28	519 ± 12	
	Ethyl laurate	10 ± 1.5	6.4 ± 0.60	9.4 ± 0.69	37 ± 2.0	26 ± 3.1	18 ± 0.38	
	Diethyl malate	369 ± 29	383 ± 37	401 ± 58	226 ± 23	98 ± 12	92 ± 5.4	
	***Sum of ethyl esters***	**5,522 ± 274 a**	**5,534 ± 31 a**	**6,106 ± 543 a**	**3,404 ± 383 a**	**2,650 ± 321 a**	**2,934 ± 49 a**	**0.03 ns**
	**Acetates**							
	Isopentyl acetate	462 ± 67	605 ± 27	265 ± 12	410 ± 32	248 ± 4.5	250 ± 14	
	Hexyl acetate	5.0 ± 0.64	3.9 ± 0.33	1.8 ± 0.19	5.3 ± 0.19	12 ± 0.77	15 ± 0.57	
	2-Phenylethyl acetate	19 ± 2.7	18 ± 1.7	13 ± 1.7	0.84 ± 0.018	10 ± 0.94	11 ± 0.53	
	***Sum of acetates***	**485 ± 68 b**	**626 ± 29 b**	**280 ± 13 a**	**416 ± 32 b**	**271 ± 4.5 a**	**277 ± 13 a**	251.34^****^
	**Fatty acids**							
	Acetic acid	229 ± 5	223 ± 30	176 ± 0.70	446 ± 63	399 ± 34	560 ± 86	
	Isobutyric acid	775 ± 80	1,043 ± 60	1,170 ± 165	1,265 ± 130	1,740 ± 104	2,080 ± 122	
	Butanoic acid	751 ± 42	856 ± 65	771 ± 101	512 ± 35	532 ± 56	557 ± 36	
	Isovaleric acid	909 ± 140	844 ± 7.6	691 ± 38	719 ± 87	678 ± 92	851 ± 45	
	Caprylic acid	2,948 ± 313	2,065 ± 68	1,206 ± 78	1,170 ± 106	763 ± 151	863 ± 46	
	Capric acid	571 ± 87	566 ± 18	419 ± 8.5	290 ± 17	166 ± 20	237 ± 22	
	***Sum of fatty acids***	**9,058 ± 769 b**	**8,116 ± 257 ab**	**5,959 ± 603 a**	**6,478 ± 515 a**	**5,278 ± 464 a**	**6,422 ± 246** **a**	51.31^****^
	**Volatile phenols**							
	Guaiacol	3.4 ± 0.37	5.0 ± 0.093	7.8 ± 1.0	1.5 ± 0.057	1.7 ± 0.071	1.5 ± 0.097	
	Eugenol	2.1 ± 0.29	1.8 ± 0.13	2.2 ± 0.33	6.9 ± 0.18	9.5 ± 1.0	9.1 ± 0.46	
	4-Ethylphenol	0.41 ± 0.045	0.65 ± 0.024	0.73 ± 0.058	0.96 ± 0.17	1.1 ± 0.13	0.57 ± 0.042	
	Syringol	6.4 ± 0.36	2.5 ± 0.11	5.0 ± 0.24	3.7 ± 0.12	6.3 ± 0.73	6.1 ± 0.14	
	Vanillin	6.5 ± 0.11	3.9 ± 0.63	4.7 ± 0.10	23 ± 0.76	63 ± 7.5	28 ± 0.68	
	Ethyl vanillate	79 ± 4.0	43 ± 2.0	70 ± 7.8	202 ± 27	137 ± 29	162 ± 13	
	Acetovanillone	40 ± 2.1	25 ± 3.9	42 ± 5.6	11 ± 0.36	12 ± 1.1	13 ± 0.57	
	***Sum of volatile phenols***	**138 ± 6.2 b**	**85 ± 3.1 a**	**135 ± 18 b**	**242 ± 27 a**	**244 ± 44 a**	**220** ± 14 **a**	**2.50 ns**
	**Lactones**							
	γ-Butyrolactone	131 ± 12	116 ± 12	119 ± 9.9	154 ± 21	280 ± 15	120 ± 8.0	
	γ-Nonalactone	8.7 ± 0.51	7.1 ± 0.44	9.3 ± 0.74	52 ± 1.0	81 ± 7.1	54 ± 2.8	
	***Sum of lactones***	**139 ± 11 a**	**123 ± 12 a**	**128 ± 11 a**	**207 ± 22 a**	**361 ± 25 b**	**173** ± 11 **a**	87.22 ^****^
	**Aldehydes**							
	Benzaldehyde	97 ± 6.7	113 ± 11	96 ± 13	161 ± 4.8	463 ± 15	355 ± 3.2	
	***Sum of aldehydes***	**97 ± 6.7 a**	**113 ± 11 a**	**96 ± 13 a**	**161 ± 4.8 a**	**463 ± 15 c**	**355 ± 3.2 b**	19.22 ^***^

Notes: **^a^** Control wine; **^b^** Wine elaborated from grapes treated under GAPs with boscalid+kresoxim-methyl; **^c^** Wine elaborated from grapes treated under GAPs with metrafenone; **^d^** Different letters within the same row indicate means significantly different at *p* < 0.01 (Fisher’s least significant difference test); ^*^^,^ ^**^^,^ ^***^^,^ ^****^ Significant *F*-values for the interaction grape variety × fungicide treatment: ^*^ for 0.1, ^**^ for 0.05, ^***^ for 0.01 or ^****^ 0.001 levels, respectively; ns = not significant.

C_6_ alcohols are formed during pre-fermentative steps (viz., harvesting, transport, crushing and pressing of grapes) when the enzymatic systems come in contact with substrates. Their synthesis has as precursors the linoleic and linolenic acids present in the grape [26]. Three C_6_ alcohols were determined in all wines, being 1-hexanol the main contributor to this group. In this case, most wines showed similar concentrations, except Tempranillo C wine and Graciano B wine which C_6_ alcohol concentrations were about 30% lower and higher, respectively, than those in their corresponding control wines. Therefore, the application of boscalid + kresoxim-methyl and metrafenone during growing grapes could induce alterations in the lipoxygenase (LOX) activity, as well as, variations in the concentrations of C_6_ alcohol precursors in grapes. Application of other active substances such as clorpyrifos, fenarimol, mancozeb, metalaxyl, penconazole and vinclozolin to Monastrell red grapes [19], or benalaxyl, iprovalicarb and pyraclostrobin to Godello white grapes [22] during the growth stage was previously found to cause no substantial change in C_6_ alcohol concentrations.

A big group of fermentative compounds are higher alcohols which are synthesized from their own precursor aminoacids by yeasts during alcoholic fermentation. There are two metabolic pathways involved in the formation of higher alcohols: the catabolic pathway of aminoacids by decarboxylation and later reduction of α-keto acids obtained by transamination of aminoacids and the anabolic pathway of aminoacids through their respective α-keto acids which are involved as intermediate in the glucidic metabolism of yeasts [27]. Chemically, higher alcohols can be aliphatic or aromatic. Aliphatic alcohols were largely (99%) isoamyl alcohols. No variation higher than 25% with respect to the control was found in the concentration of aliphatic alcohols in Tempranillo and Graciano B-C wines. Besides, this variability was not statistically significant, therefore, it may be assumed that the assimilation of the aminoacids leucine and isoleucine, the precursors of 3-methyl-1-butanol and 2-methyl-1-butanol, respectively, was not affected by the application of fungicides [28]. A similar outcome was previously found in wines from Godello white grapes treated under GAPs [21] or CAPs [19] with other new-generation fungicides. By contrast, application of quinoxyfen and trifloxystrobin under GAPs significantly increased the 3-methyl-1-butanol concentration of Monastrell red wines [20].

2-Phenylethanol was the greatest contributor to aromatic alcohols, and hence, to differences in their total concentration. Treatments with metrafenone (wine C) seemingly altered the concentration of these volatile compounds in Tempranillo and Graciano-based wines, which was decreased by 45%; whereas boscalid+kresoxim-methyl only appeared to induce substantial changes in Graciano wines, reducing the concentration of aromatic alcohols by 45%. Although aromatic alcohols are mainly synthesized by yeasts during alcoholic fermentation, they are already in grapes [29]. Both boscalid + kresoxim-methyl and metrafenone are systemic fungicides against powdery mildew and were applied under GAPs to the vineyard; this may have altered grape metabolism by diminishing the production of aminoacids such as phenylalanine, which is required for the synthesis of 2-phenylethanol by yeasts [30]. This is consistent with previous results of González-Rodríguez *et al.* [21], who found significantly decreased 2-phenylethanol concentrations in wines from Godello white grapes containing metiram, pyraclostrobin, benalaxyl, cymoxanil and folpet residues; nevertheless, most fungicides studied so far (*viz*., cyprodinil, famaxadone, fenhexamid, fludioxonil, fluquinconazole, quinoxyfen, pyrimethanil and trifloxystrobin) produced an increase [20,31].

Esters in wine are synthetized enzymatically by yeasts during alcoholic fermentation. According to their structure, esters can be classified as ethyl esters and acetates. Within ethyl esters, ethyl lactate was the most abundant in both Tempranillo and Graciano wines. The overall concentrations of ethyl esters were similar in virtually all Tempranillo wines, meanwhile in Graciano wines, their concentrations were slightly lower in wines B and C than in A (control wine), but the differences never exceeded 25%. Similarly, no significant difference was previously found by González-Álvarez *et al.* [22] in Godello white wines obtained from grapes treated under CAPs with mandipropamid, famoxadone and valifenalate. However, some authors have suggested that the concentration of ethyl esters may be affected by the nature and concentration of fungicide residues [6,7,17,19,20,21,24]. Concerning the concentration of acetates, treatments wiht boscalid + kresoxim-methyl and metrafenone seemingly altered the synthesis of these fermentative volatile compounds, as significant decreases (above 25%) with respect to control wines were found in all wines elaborated from treated grapes, except wine B from Tempranillo grapes.

Fatty acids, together with esters and higher alcohols, are the main indicators of alcoholic fermentation. Only the application of metrafenone on Tempranillo grapes appeared to influence the total concentration of fatty acids in the obtained wines, reducing their content about 35%, whereas differences from the control wines were less than 25% for the rest of treatments. There were, however, remarkable differences in the concentration of individual acids. Thus, the concentration of isobutyric acid was higher in all wines elaborated from treated grapes and those of caproic and caprylic acids were lower than in the control wines. The last two acids, along with capric acid, are deemed quality enhancing factors in winemaking provided their concentrations do not exceed 20 mg/L [32]. Therefore, their lower concentrations suggest that the application of boscalid+kresoxim-methyl and metrafenone may reduce the quality of Tempranillo and Graciano red wines. Oliva *et al.* [20] also found significantly decreased concentrations of caproic and caprylic acids in wines from Monastrell red grapes treated with other fungicides including quinoxyfen, famoxadone, trifloxystrobin, fluquinconzole and fenhexamide.

The total concentrations of volatile phenols were similar in all Graciano wines. Regarding to Tempranillo wines, the application of boscalid + kresoxim-methyl seemed to induce a substantial reduction in the concentration of volatile phenols (about 40%). So far, only González-Álvarez *et al.* [22] and Noguerol-Pato *et al.* [24] examined the effect of fungicides on the concentrations of volatile phenols. Specifically, the former found mandipropamid, cyazofamid and famoxadone to decrease such concentrations in Godello white wines, and valifenalate to have no effect on them; whereas the latter showed significantly lower concentrations of volatile phenols in Tempranillo wines when mepanipyrim and fenhexamid were added to musts.

Lactones are produced during alcoholic fermentation and result from an internal esterification reaction between an acid function and an alcohol function in the same molecule [33]. All Tempranillo-based wines exhibited nearly identical concentrations of lactones irrespective of fungicide treatment. On the other hand, Graciano wine B showed a significantly higher total concentration of lactones (about 75%) than wine A (control wine). This result suggests that the treatment with boscalid + kresoxim-methyl may cause a metabolic disorder in yeast activity leading to increased lactone production.

Benzaldehyde was the sole compound detected in the aldehydes family. They are barely detected as aromatic constituents of wines since they can be reduced to their respective alcohols during alcoholic fermentation [34]. Tempranillo-based wines showed comparable concentrations of benzaldehyde. However, Graciano B and C wines contained much higher (120%–180%) benzaldehyde concentrations than wine A (control) which can be attributed to an alteration on the activity of the alcohol dehydrogenase enzymes which are responsible for the reduction of aldehydes to their respective alcohols.

Some authors have assessed the effects of the application of resistance inducers or elicitors in vineyard on wine aroma compounds. These compounds (viz. chitosan, benzothiadiazole and methyl jasmonate) are a class of products able to elicit the plant defence mechanisms against pathogens, incurring lower toxicological risks than conventional agrochemicals. However, as in the case of conventional fungicides, elicitors lead to changes in the volatiles content of grapes and wines, as well as in the sensory attributes of wines made from grapes treated with these products [35,36,37]. Gómez-Plaza *et al.* [35] observed higher levels of volatile compounds in Monastrell grapes treated with benzothiadiazole and methyl jasmonate, especially terpenes and norisoprenoids in benzothiadiazole-treated grapes. Besides, wines obtained from treated grapes also showed higher levels of these volatile compounds leading to more valuable aromatic wines. On the other hand, Vitalini *et al.* [37] found that, compared with conventional fungicides, the application of chitosan and benzothiadiazole in vineyard induced specific effects on the volatile content of wines. In particular, benzothiadiazole increased total acetals and esters, while chitosan raised the level of total acetals and alcohols in Groppello Gentile red wines.

### 2.2. Effect of Application under GAPS of Boscalid + Kresoxim-Methyl and Metrafenone on the Aromatic Profile of Tempranillo and Graciano Red Wines

As it was previously stated, substantial variations in the concentration of some volatile compounds were observed in Tempranillo and Graciano red wines when treatments with boscalid + kresoxim-methyl and metrafenone were carried out under GAPs during the vine growth. Additionally, such changes can lead to alterations on the aroma profile of the obtained wines.

In order to assess potential modifications on the final aroma of wines, the OAV of each volatile compound was calculated. However, using individual OAVs to establish the aromatic profile of wines makes rather difficult to interpret the data and to draw clear-cut conclusions. Thus, volatile compounds with similar odour descriptors were grouped into seven odorant series defined by a generic descriptor (Table 2). The total OAV of each odorant series was calculated by adding the OAV of every volatile compound belonging to a particular series. In that way, evaluating the aromatic profile of the wines can be done in simpler terms.

**Figure 2:**
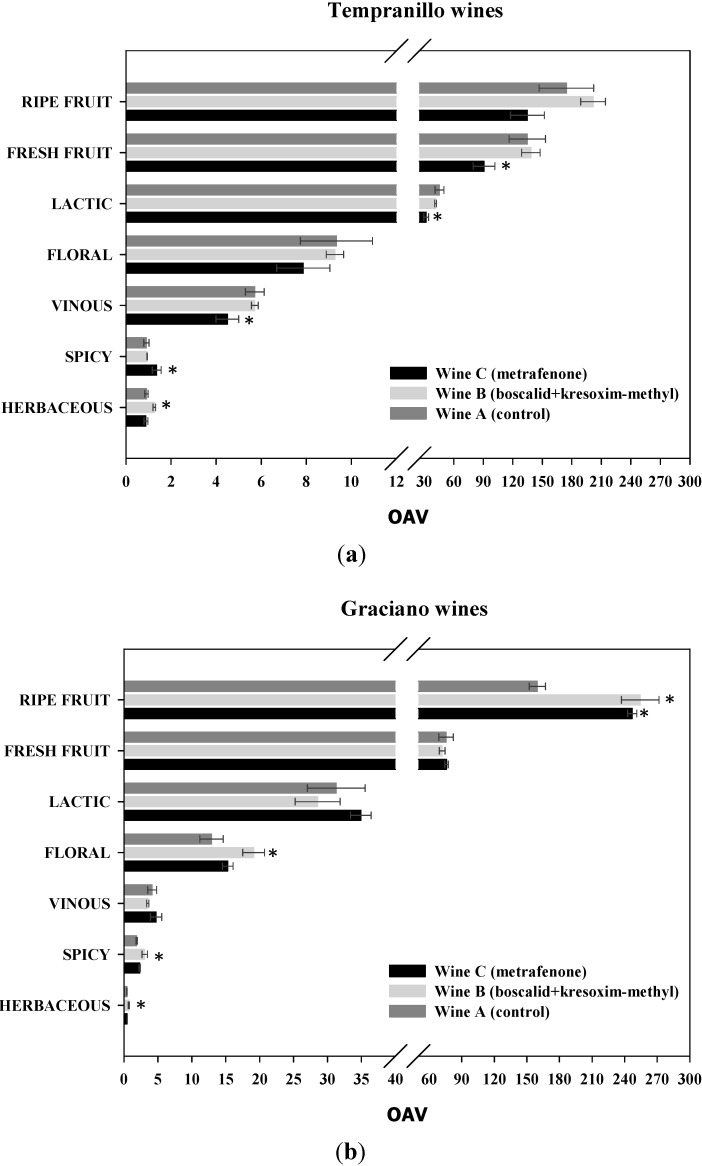
Aromatic profile of monovarietal red wines according to their odorant series: (**a**) Tempranillo-based wines and (**b**) Graciano-based wines. Significant differences at *p* < 0.05 (Fisher’s least significant difference test) with respect to control wines were pointed out with an asterisk (*).

Figure 2a,b show the contribution of each odorant series to the aroma profile of Tempranillo and Graciano monovarietal red wines. According to their OAVs, ripe fruit and fresh fruit were the most important aromatic attributes in all red wines, and, after them, lactic, floral, vinous, spicy and herbaceous nuances. In general, Graciano red wines were richer in ripe fruit and floral notes, whereas those from Tempranillo grape variety were in fresh fruit, lactic and vinous odorant series. OAVs for herbaceous odorant series, hardly reached the unit in most wines, so the contribution of this attribute to the overall aroma profile of wines was of minor importance.

**Figure 3:**
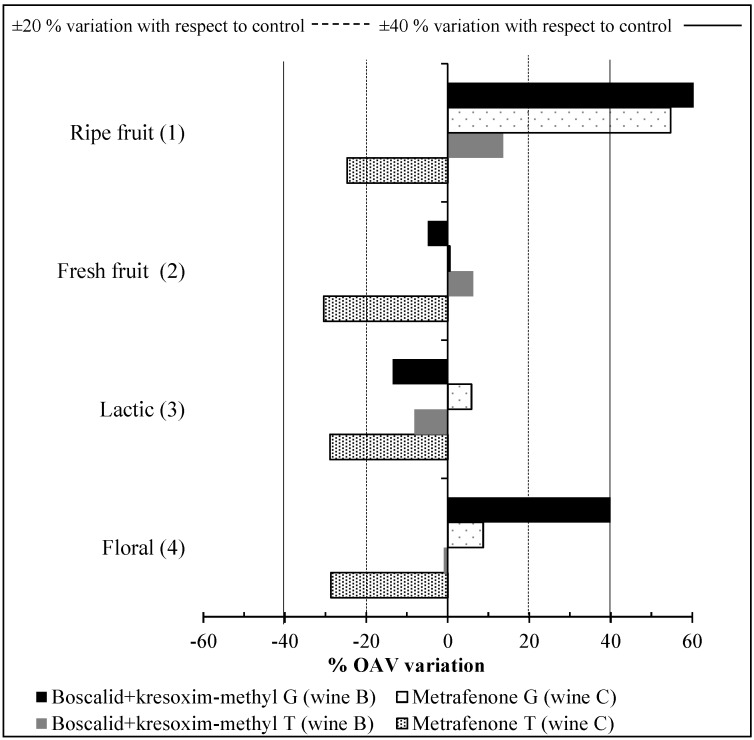
Percentages of variation with respect to the control wine on the OAV of monovarietal red wines (G, Graciano; T, Tempranillo) by effect of boscalid + kresoxim-methyl and metrafenone (in brackets the number of the odorant series as reported in Table 2).

The effect of the application of boscalid + kresoxim-methyl and metrafenone on the aroma profile of Tempranillo and Graciano wines is depicted on Figure 3 by calculating the percent changes in the OAVs for the main odorant series by effect of each active substance.

**Table 2:** Classification of volatile compounds into odourant series according to their odour descriptors.

Volatile Compounds	Odour Threshold (μg/L)	Odour Descriptors	Odourant Series ^a^
(±)-Linalol	15 ^b^	Orange flowers, citrus	2; 4
α-Terpineol	250 ^c^	Lilac	4
(±)-β-Citronellol	100 ^d^	Rose, citrus	2; 4
Geraniol	30 ^b^	Geranium, rose, citric	2; 4
**C_13_-norisoprenoids**			
β-Damascenone	0.05 ^b^	Dry plum	1
β-Ionone	0.09 ^c^	Violets	4
**C_6_ alcohols**			
1-Hexanol	8000 ^b^	Grass	7
*trans*-3-Hexen-1-ol	1000 ^f^	Green	7
*cis*-3-Hexen-1-ol	400 ^b^	Grass	7
**Aromatic alcohols**			
Benzyl alcohol	200,000 ^d^	Walnut, fruity	2
2-Phenylethanol	10,000 ^b^	Rose	4
**Aliphatic alcohols**			
2-Methyl-1-propanol	40,000 ^d^	Alcohol	5
1-Butanol	150,000 ^d^	Alcohol	5
Isomayl alcohols	30,000 ^b^	Alcohol	5
1-Octanol	10,000 ^e^	Rose, jasmine, citrus	2; 4
**Acetates**			
Isopentyl acetate	30 ^c^	Banana	1
Hexyl acetate	1500 ^c^	Apple, pear, banana	1; 2
2-Phenylethyl acetate	250 ^b^	Rose	4
**Esters**			
Ethyl caproate	14 ^d^	Green apple, banana	1; 2
Ethyl lactate	154,636 ^c^	Strawberry, raspberry, buttery	2; 3
Ethyl caprylate	5 ^d^	Pineapple, strawberry	1; 2
Ethyl 3-hydroxybutyrate	20,000 ^d^	Grape-like	2; 5
Ethyl decanoate	200 ^c^	Sweet, fruity	1
Diethyl succinate	200,000 ^d^	Wine-like	5
Ethyl laurate	500 ^f^	Fruity, floral	2; 4
Diethyl malate	760,000 ^d^	Over-ripe, peach	1
**Fatty acids**			
Acetic acid	200,000 ^b^	Pungent, vinegar	3
Isobutyric acid	2300 ^d^	Rancid, butter, cheese	3
Butanoic acid	173 ^d^	Rancid, butter, sweat	3
Isovaleric acid	33 ^c^	Acid, rancid	3
Caproic acid	420 ^c^	Sweat	3
Caprylic acid	500 ^c^	Sweat, cheese	3
Capric acid	1000 ^d^	Rancid fat	3
**Volatile phenols**			
Guaiacol	10 ^c^	Sweet, smoky	6
Eugenol	6 ^c^	Clove, liquorice	6
4-Ethylphenol	450 ^g^	Phenolic, bitumen	6
Syringol	570 ^g^	Smoky	6
Vanillin	60 ^d^	Vanilla	6
Ethyl vanillate	990 ^d^	Honey, vanillin	6
Acetovanillone	1000 ^d^	Vanilla, clove	6
**Lactones**			
γ-Butyrolactone	35 ^c^	Coconut	1
γ-Nonalactone	30 ^c^	Coconut	1
**Aldehydes**			
Benzaldehyde	350 ^c^	Sweet, cherry	1; 2

Notes: **^a^** 1 = Ripe fruit; 2 = Fresh fruit; 3 = Lactic; 4 = Floral; 5 = Vinous; 6 = Spicy; 7 = Herbaceous; **^b^** [38]; **^c^** [39]; **^d^** [32]; **^e^** [40]; **^f^** [41]; **^g^** [42].

C_13_-norisoprenoids and ethyl esters were the main contributors to ripe fruit nuances. However, the volatile compounds with more weight in this odorant series depended on the grape variety. β-Damascenone, with a dry plum individual descriptor, was the chief aroma in Graciano red wines, while ethyl caprylate, with a pineapple descriptor, was the most important odorant attribute in quantitative terms in Tempranillo wines. Therefore, the changes in the aroma profile of wines elaborated from treated grapes arose from variations in the concentration of these two volatile compounds. The presence of fungicide residues in Graciano red wines (Table 3) considerably boosted ripe fruit notes, however this increase is not always associated with high quality wines, since a balanced aroma is a more appreciate attribute on consumers’ overall perception of wine quality. By contrast, wine C from Tempranillo red grapes (treated with metrafenone) exhibited a decrease greater than 20% by comparison with the control wine as a result of a reduction in the concentration of isopentyl acetate (banana descriptor) and ethyl caprylate (pineapple descriptor).

**Table 3:** Commercial formulations used in open-field treatments.

Wine	A ^a^	B	C
**Commercial name**	-	Collis	Vivando
**Fungicide formulation**	-	20% boscalid + 10% kresoxim-methyl	50% metrafenone
**Fungal disease**	-	Grey mould and powdery mildew	Powdery mildew
**Fungicide concetration in Tempranillo grapes (mg/Kg) ^b^**	-	15 + 5.4	2.8
**Fungicide concetration in Graciano grapes (mg/Kg) ^b^**	-	22 + 8.2	1.5
**Residual concentrations of fungicides in Tempranillo wines (μg/L)^b^**	-	1087 + 388	197
**Residual concentrations of fungicides in Graciano wines (μg/L) ^b^**	-	1548 + 586	107

Notes: **^a^** Control treatment; **^b^** Concentrations were determined following the method proposed by Lagunas-Allué *et al.* [43].

The presence of residual metrafenone in Tempranillo wines (Table 3) reduced the OAV for the fresh fruit series by more than 20% with respect to the control wines. These changes were mainly the result of a drop in the OAV for ethyl caproate and ethyl caprylate, which contribute to green apple and strawberry notes, respectively.

The lactic odorant series in Graciano and Tempranillo red wines exhibited no significant difference from the control wines except for Tempranillo wine C, which was obtained from metrafenone-treated grapes and exhibited a decrease by 28% in the OAV for this series. Since all fatty acids belong to the lactic odorant series, this is consistent with what happened with the total concentration of fatty acids, which only showed higher variation than 25% in Tempranillo wine C. Although the individual odor descriptors of each fatty acid are unpleasant, the contribution of this chemical family to the overall aroma of wines is related to a rounded, smooth taste [44]. Therefore, the decrease of this series in Tempranillo C wine may reduce its aromatic quality.

Floral nuances were highly reduced in Tempranillo wine C, but increased in Graciano wine B. The floral series comprised violet (β-ionone) and rose notes (2-phenylethanol) mainly. Both Graciano and Tempranillo wines elaborated from treated grapes exhibited a decrease in rose scent as a result of the decline of 2-phenylethanol concentration; however, the markedly increased violet nuances of Graciano wine B countered the decline in rose notes and led to a wine with a significantly higher OAV for the floral series.

All aliphatic alcohols are included in the vinous odorant series and they constitute the basic aroma fraction of all wines. The presence of residues of the fungicides was found to have a slight effect on the vinous character of Graciano and Tempranillo red wines. Overall, the vinous series was thus unaffected by application of the fungicides.

Despite being less important in quantitative terms, spicy notes contribute to wine complexity and is typically used in the aromatic characterization of wines [45,46], so that any loss of these compounds is undesirable with a view to obtaining high-quality wines. Significant differences were observed for the spicy odorant series in Tempranillo wine C and Graciano wine B. It is worth mentioning that the residues of metrafenone in Tempranillo wines seemed to increase the OAV of the spicy series above 1, which may set the difference in its sensory perception. Finally, the variations observed in the herbaceous odorant series, which only included C_6_ alcohols, were no qualitatively significant as all wines exhibited OAVs lower than 1, except in Tempranillo B wine.

## 3. Experimental Section

### 3.1. Fungicide Treatments, Winemaking Process and Wine Samples

Trials were conducted in an experimental vineyard located in Aldeanueva de Ebro, La Rioja (N Spain), in the Qualified Designation of Origin “Rioja”. The vineyard, which produces red grapes of the *Vitis vinifera* cv. Graciano and cv. Tempranillo, was split into three experimental plots of six rows with 40–50 vines each (A to C). The gap between rows and grapevines was 2.6 and 1.2 m, respectively. Plot A was left untreated for use as a control; plots B and C were treated under GAPs (*i.e.*, using the doses recommended by the manufacturer and keeping the pre-harvest interval, PHI) with Collis and Vivando, respectively. Table 3 summarizes the characteristics of the commercial formulations used in the different treatments, as well as the fungicide concentrations determined in harvested grapes and final wines.

In order to avoid contamination, only grapes from the two central rows were harvested in September 2012. After that, grapes from each plot A–C were subjected to identical vinifications in the experimental cellar of the University of La Rioja as follows: grapes were crushed, destemmed and placed in a metallic fermentation vessel (40 L) which was supplied with SO_2_ at 50 mg/L. During the alcoholic fermentation–maceration the temperature was kept between 17–21 °C and it took 14 days. At the end of the process, the wine was strained off and transferred to a metallic vessel where it was supplied with SO_2_ at 30 mg/L. Prior to bottling, a cold clarification step was carried out. In Table 4 the general parameters (pH, titratable and volatile acidity, alcoholic content, total and free SO_2_) of the final wines are shown.

### 3.2. Chemicals

The solvents used included dichloromethane, methanol and water (HPLC quality) which were purchased from Sigma-Aldrich (Steinheim, Germany), and ethanol absolute (HPLC grade) which was acquired from Scharlau (Barcelona, Spain); anhydrous sodium sulphate for residue analysis was obtained from Panreac (Barcelona, Spain). The sorbent material used for solid-phase extraction (SPE) was Strata-X, 33 μm polymeric reversed phase from Phenomenex (Torrance, CA, USA). Small apparatus such as an Ultrasons-H ultrasound bath (JP Selecta, Barcelona, Spain), a Reax Top vortex (Heidolph, Schwabach, Germany), a Visiprep SPE Vacuum Manifold (Supelco, Bellefonte, PA, USA) and a Turbo Vap LV evaporator (Caliper Life Sciences, Hopkinton, MA, USA) were also used. Standards for determining volatile compounds were purchased from Sigma-Aldrich and used to prepare stock standard solutions in ethanol according to Noguerol-Pato *et al.* procedure [47].

**Table 4:** General parameters of Tempranillo and Graciano final wines.

General Parameter	Tempranillo Wines			Graciano Wines		
	Wine A	Wine B	Wine C	Wine A	Wine B	Wine C
**Total titratable acidity ^a^ (g tartaric acid/L)**	5.7 ± 0.5	6.2 ± 0.5	5.3 ± 0.5	5.5 ± 0.5	6.8 ± 0.5	5.8 ± 0.5
**Total volatile acidity** **^a^ (g acetic acid/L)**	0.5 ± 0.11	0.42 ± 0.11	0.62 ± 0.11	0.42 ± 0.11	0.45 ± 0.11	0.44 ± 0.11
**Reducing sugars ^b^ (glucose+fructose) (g/L)**	<0.2	<0.2	<0.2	<0.2	<0.2	<0.2
**Ethanol ^a^ (%, v/v)**	14.3 ± 0.2	13.9 ± 0.2	14.5 ± 0.2	14.2 ± 0.2	13.9 ± 0.2	14.5 ± 0.2
**pH ^a^**	3.67 ± 0.12	3.56 ± 0.12	3.91 ± 0.12	3.51 ± 0.12	3.35 ± 0.12	3.69 ± 0.12
**Free SO_2_^c^ (mg/L)**	<10	<10	<10	<10	<10	<10
**Total SO_2_^c^ (mg/L)**	11	13	<10	<10	<10	<10

^a^ These parameters were determined by Fourier transform infrared spectroscopy (FTIR); ^b^ These parameters were determined by enzymatic method; ^c^ These parameters were determined using a continuous segmented flow analyzer.

### 3.3. Extraction, Separation and Identification Procedures

A solid-phase extraction (SPE) system was used for concentration and clean-up of volatile compounds, using the method developed by González-Álvarez *et al.* [22] with slight modifications proposed by Noguerol-Pato *et al.* [24]. Wine samples (50 mL) containing 20 μL of 4-nonanol (50 mg/L in ethanol) as surrogate standard were loaded in a Strata-X cartridge (500 mg, 6 mL size) previously conditioned with methanol (17 mL) and water (20 mL at pH 3.7). A cleaning step with water (20 mL at pH 3.7) was performed after the sample loading. Subsequently, the sorbent was dried by passing N_2_ for 45 min, and then volatiles were eluted with dichloromethane (10 mL). The eluate was dried over anhydrous sodium sulphate, concentrated to a volume <1 mL under a N_2_ stream, enriched with 20 μL of 2-octanol (100 mg/L in ethanol) as internal standard and made up to 1 mL with dichloromethane prior to gas chromatographic analysis.

Volatile compounds were separated and identified on a Trace GC instrument equipped with a PolarisQ ion trap mass selective detector (ITMS) that was furnished with an AS 3000 automatic injector from Thermo Finnigan (Rodano, Italy) and interfaced to a PC computer running the software Xcalibur 1.4, from Thermo Scientific. Chromatographic separations were done on a TR-WAX MS polyethylene glycol capillary column (60 m × 0.25 mm ID, 0.25 μm film thickness). The oven temperature programme was as follows: 40 °C for 2 min; 3 °C/min ramp to 145 °C; 2 °C/min ramp to 158 °C (held for 2 min); 3 °C/min ramp to 210 °C and 4 °C/min ramp to 250 °C (held for 2 min). The carrier gas, helium, was circulated at 1 mL/min in the constant flow mode and the injected volume was 2 μL. A PTV injector was used in the splitless mode (splitless time: 0.75 min) using the following temperature programme: 45 °C for 0.05 min; 14.5 °C/min ramp to 200 °C (held for 60 min). The transfer line temperature was 250 °C and the ion-trap manifold temperature 200 °C. The ion energy for electron impact (EI) was 70 eV.

Identification and confirmation of the volatile compounds were achieved by comparing the GC retention times and mass spectra over the mass range 35–300 amu for the samples with those for pure standards analyzed under the same conditions. Quantification was performed by choosing specific *m/z* values of each volatile compound from the *full-scan* mode. The concentrations of volatile compounds were determined by the internal standard method; calibration curves with eight concentration levels in duplicate were used and red wine recoveries were applied to guarantee reliable results. Recoveries ranged from 80% to 100% for most of the volatiles and repeatability, expressed as RSD%, was less than 15% for most of the volatiles (data not shown).

### 3.4. Odour Activity Values

The odour activity value (OAV) for each volatile compound was calculated from the equation *OAV = c/t*, where *c* is the total concentration of the compound concerned in the wine and *t* its odour threshold value [38,48,49]. Volatile compounds with similar odour descriptors were classified into seven odorant series (ripe fruit, fresh fruit, lactic, floral, vinous, spicy and herbaceous) according to Noguerol-Pato *et al.* [24]. The overall OAVs for each series were determined by combining the values of their individual volatile compounds.

### 3.5. Statistical Treatment

Significant differences among wine samples from the same grape variety were assessed by one-way ANOVA at 99% confidence level, for the total concentration of volatile compounds, and at 95% confidence level, for the OAVs of the odorant series. A Fisher’s least significant difference (LSD) test, at 99% confidence level, was used to compare among means. Differences were considered significant at *p* < 0.01 and represented by different letters. In order to examine the interdependences between fungicide treatments and grape variety, two-way ANOVA was performed for the total concentration of volatile compounds. Statistical analyses were performed with the software package Statgraphics Plus V. 5.1 (Manugistics, Rockville, MD, USA).

## 4. Conclusions

In summary, phytosanitary treatments with the fungicides boscalid + kresoxim-methyl and metrafenone may have induced changes in the concentrations of volatile compounds and, hence, in the aroma profile of Graciano and Tempranillo red wines in a variety-dependent manner. The concentrations of volatile compounds in Tempranillo-based wines showed different behavior depending on the applied fungicide. On the other hand, Graciano red wines responded in a similar way to the application of boscalid + kresoxim-methyl and metrafenone, but, in general terms, the presence of residues of these fungicides in the wines increased the concentrations of varietal compounds, particularly C_13_-norisoprenoids. These changes in volatile composition altered the aroma profile of the studied wines. Thus, the presence of boscalid + kresoxim-methyl residues increased ripe fruit and floral nuances in Graciano red wines, mainly as a result of enhanced dry plum and violet notes from β-damascenone and β-ionone, respectively. Besides, residues of metrafenone also produced an increase in the ripe fruit odorant series. By contrast, Tempranillo red wines were greatly affected by the presence of metrafenone residues, which reduced virtually all odorant series. 
