# Correction: Noguerol-Pato, R., *et al*. Effect on the Aroma Profile of *Graciano* and *Tempranillo* Red Wines of the Application of Two Antifungal Treatments onto Vines. *Molecules* 2014, *19*, 12173-12193

**DOI:** 10.3390/molecules191117422

**Published:** 2014-10-29

**Authors:** Raquel Noguerol-Pato, Thais Sieiro-Sampedro, Carmen González-Barreiro, Beatriz Cancho-Grande, Jesús Simal-Gándara

**Affiliations:** Group of Nutrition and Bromatology, Department of Analytical and Food Chemistry, Faculty of Food Science and Technology, University of Vigo, Ourense E-32004, Spain

The authors wish to make the following correction to paper [1], doi:10.3390/molecules190812173, website: http://www.mdpi.com/1420-3049/19/8/12173.

The author name “Thais Sieiro-Sampredro” should be “Thais Sieiro-Sampedro”.

We have detected an error in the results expression units, and the decimal “.” has to be moved 1 position, resulting in a factor of 10. The corrected data were provided in the new version of the Table 3 (under column B). 

**Table 3:** Commercial formulations used in open-field treatments.

Wine	A ^a^	B	C
**Commercial name**	-	Collis	Vivando
**Fungicide formulation**	-	20% boscalid + 10% kresoxim-methyl	50% metrafenone
**Fungal disease**	-	Grey mold and powdery mildew	Powdery mildew
**Fungicide concentration in Tempranillo grapes (mg/Kg) ^b^**	-	1.5 + 0.54	2.8
**Fungicide concentration in Graciano grapes (mg/Kg) ^b^**	-	2.2 + 0.82	1.5
**Residual concentrations of fungicides in Tempranillo wines (µg/L) ^b^**	-	109 + 39	197
**Residual concentrations of fungicides in Graciano wines (µg/L) ^b^**	-	155 + 59	107

Notes: ^a^ Control treatment; ^b^ Concentrations were determined following the method proposed by Lagunas-Allué *et al.* [43].
